# Supplementary material for: Availability, prices and affordability of essential medicines: A cross-sectional survey in Hanam province, Vietnam
Source: PLoS One. 2021 Nov 18;16(11):e0260142. doi: 10.1371/journal.pone.0260142 (PMC8601520; doi:10.1371/journal.pone.0260142)
Supplement: S1 Table — (DOCX) [file pone.0260142.s002.docx]

**S1 Table. Surveyed areas and the number of health facilities from which data were collected in this research**

| **Areas** | **Public sector** | | **Private sector** | **Total** |
| --- | --- | --- | --- | --- |
|  | **Hospital** | **Health center/station** | **Medicine outlets** |  |
| Phuly city | 3 | 2 | 5 | **10** |
| Duytien district | 1 | 4 | 6 | **11** |
| Lynhan district | 1 | 4 | 5 | **10** |
| Kimbang district | 1 | 4 | 6 | **11** |
| Binhluc district | 1 | 4 | 7 | **12** |
| Thanhliem district | 1 | 4 | 6 | **11** |
| **Total** | **8** | **22** | **35** | **65** |
