# Supplementary material for: Availability, prices and affordability of essential medicines: A cross-sectional survey in Hanam province, Vietnam
Source: PLoS One. 2021 Nov 18;16(11):e0260142. doi: 10.1371/journal.pone.0260142 (PMC8601520; doi:10.1371/journal.pone.0260142)
Supplement: S2 Table — (DOCX) [file pone.0260142.s003.docx]

**S2 Table. The list of surveyed medicines**

| **No** | **Medicine** | | **NEML** | **Class of medicine** |
| --- | --- | --- | --- | --- |
|  | **Name, strength** | **Unit** |  |  |
|  | *Core medicines* |  |  | Anti-inflammatory Agent |
| 1 | Amitriptyline 25 mg | tablet/capsule | yes | Antidepressant |
| 2 | Amoxicillin 500 mg | tablet/capsule | yes | Antibacterial |
| 3 | Bisoprolol 5 mg | tablet/capsule | no | Cardiovascular agent |
| 4 | Captopril 25 mg | tablet/capsule | yes | Cardiovascular agent |
| 5 | Ceftriaxone 1 g | vial | yes | Antibacterial |
| 6 | Ciprofloxacin 500 mg | tablet/capsule | yes | Antibacterial |
| 7 | Co-trimoxazole 40 + 8 mg/ml | ml | yes | Antibacterial |
| 8 | Diazepam 5 mg | tablet/capsule | yes | Anxiolytic |
| 9 | Diclofenac 50 mg | tablet/capsule | yes | Anti-inflammatory Agent |
| 10 | Metformin 500 mg | tablet/capsule | yes | Antidiabetic Agent |
| 11 | Omeprazole 20 mg | tablet/capsule | yes | Proton Pump Inhibitor |
| 12 | Paracetamol 24 mg/ml | ml | no | Antipyretic/Analgesic |
| 13 | Salbutamol 100 mcg/dose | dose | yes | Bronchodilator |
| 14 | Simvastatin 20 mg | tablet/capsule | yes | Dyslipidemic |
|  | *Supplementary medicines* |  |  |  |
| 15 | Albendazole 200 mg (chewable) | tablet/capsule | yes | Antiparasitic |
| 16 | Amlodipine 5 mg | tablet/capsule | yes | Cardiovascular agent |
| 17 | Atorvastatin 20 mg | tablet/capsule | yes | Dyslipidemic |
| 18 | Cefalexin 500 mg | tablet/capsule | yes | Antibacterial |
| 19 | Co-trimoxazole 400 + 80 mg | tablet/capsule | yes | Antibacterial |
| 20 | Enalapril 5 mg | tablet/capsule | yes | Cardiovascular agent |
| 21 | Furosemide 40 mg | tablet/capsule | yes | Duretic |
| 22 | Gliclazide 30 mg | tablet/capsule | yes | Antidiabetic agent |
| 23 | Ibuprofen 400 mg | tablet/capsule | yes | Anti-inflammatory Agent |
| 24 | Insulin 100 IU/ml | vial | yes | Blood Glucose Regulators |
| 25 | Mebendazole 500 mg (chewable) | tablet/capsule | yes | Antiparasitic |
| 26 | Metronidazole 250 mg | tablet/capsule | yes | Antibacterial |
| 27 | Morphine 10 mg/ml | vial | yes | Analgesic |
| 28 | Nifedipine retard 20 mg | tablet/capsule | yes | Cardiovascular agent |
| 29 | ORS powder (for 1 litre) | sachet | yes | Rehydration |
| 30 | Paracetamol 500 mg | tablet/capsule | yes | Antipyretic/Analgesic |
| *NEML: National Essential Medicine List, IU: International unit*  *ORS: Oral rehydration salt/solution* | | | | |
