# Supplementary material for: Availability, prices and affordability of essential medicines: A cross-sectional survey in Hanam province, Vietnam
Source: PLoS One. 2021 Nov 18;16(11):e0260142. doi: 10.1371/journal.pone.0260142 (PMC8601520; doi:10.1371/journal.pone.0260142)
Supplement: S3 Table — (DOCX) [file pone.0260142.s004.docx]

**S3 Table. Median MPRs for eight medicines found as both product types in the private sector**

| **Type** | **Median MPRs** | | | | |
| --- | --- | --- | --- | --- | --- |
|  | **Min** | **25^th^ percentile** | **Median** | **75^th^ percentile** | **Max** |
| Originator brand | 2.21 | 3.89 | 8.44 | 11.69 | 38.47 |
| Lowest price generic | 0.76 | 1.28 | 1.87 | 2.20 | 10.21 |
| *MPR: Median Price Ratio* | | | | | |
