# Supplementary material for: Availability, prices and affordability of essential medicines: A cross-sectional survey in Hanam province, Vietnam
Source: PLoS One. 2021 Nov 18;16(11):e0260142. doi: 10.1371/journal.pone.0260142 (PMC8601520; doi:10.1371/journal.pone.0260142)
Supplement: S4 Table — (DOCX) [file pone.0260142.s005.docx]

**S4 Table. Median MPRs for medicines found in both public and private sectors**

| **Product type** | **Median MPR**  **Public sector** | **Median MPR**  **Private sector** | **% difference private to public** |
| --- | --- | --- | --- |
| Originator brand  (n = 0 medicines) | - | - | - |
| Lowest price generic  (n = 18 medicines) | 0.95 | 1.28 | 35.4% |
| *MPR: Median Price Ratio* | | | |
