# Supplementary material for: Availability, prices and affordability of essential medicines: A cross-sectional survey in Hanam province, Vietnam
Source: PLoS One. 2021 Nov 18;16(11):e0260142. doi: 10.1371/journal.pone.0260142 (PMC8601520; doi:10.1371/journal.pone.0260142)
Supplement: Attachment — Submitted filename: Reviewers Comments for the editor.docx [file pone.0260142.s006.docx]

Reviewer’s Comment_PONE-D-21-15309

Thank you for the opportunity to review this scholarly manuscript. The erudite disposition of the authors is commendable.

The paper has claimed that the availability of OBs was significantly low in both public and private sectors. Generic medicines were the predominant product type available in both public and private sectors. The mean availability of LPGs was fairly high but still lower than the benchmark of WHO. The prices of OBs were high and were unaffordable in private drug stores. LPGs in both sectors were sold to patients at reasonable prices compared to International retail prices. These claims are properly placed in the context of available literature and the literature were fairly treated. Also, the data and analysis fully support the claim.

A few minor remarks were noted. These would require the authors’ attention.

Abstract

- Result (line 33): insert the exact p value.

Introduction

Line 50: Insert reference.

Line 66: ………There are several studies on medicine prices, availability and affordability conducted in Vietnam [11, 12]. Some small scale studies were carried out but not published……….

**What then is the gap in knowledge that this current study aimed to fill? Does this suggest that these studies were not on essential medicines**

Materials and methods

Lines 86/87: ………A licensed private drugstore closest to each of the selected public facilities was selected for the private sector………

**The WHO/HAI methodology also provides for survey of 'Other' pharmacies which include those domiciled in private/organization owned hospitals. Does the province have these categories of hospitals? if yes, why were they not included in the survey? if no, a brief description of the health facility set up in Hanam would suffice.**

Line 90**: write ‘30’ in words**

Results:

Table 3: Urban (11 drug stores) versus rural (24 drug stores) comparison

**Does this suggest that most of the districts surveyed were in the rural area? A brief sentence regarding the geography of these areas should be added in the methods section**

S1 Table

**It is suggested that these medicines be group according to their class of drug. For example: Antibiotics, Psychotropic, Narcotics, Anti-inflammatory, Analgesics, Asthma medicines, Antihypertensive, Antidaibetic etc**

Discussion

Line 215: …….fairly high (public sector 63.2%, private sector 47.9%)……….

**Recast to reflect that availability of LPGs in private sector was fairly high but that in public sector was low (47.9%)**

Line 283: Vietnamese government has implemented many national policies to increase the availability reduce medicine prices

**The authors should stress the need for the enforcement of the price regulation in the private sector. Despite that fact that prices are generally lower than those of other countries, the private sector still sells OBs higher than the LPGs to the tune of 351.34%. Unless the LPGs are of reasonable quality and efficacy in treatment of the particular disease. Are there concerns for substandard medicines in Hanam?**
