# Supplementary material for: Availability, prices and affordability of essential medicines: A cross-sectional survey in Hanam province, Vietnam
Source: PLoS One. 2021 Nov 18;16(11):e0260142. doi: 10.1371/journal.pone.0260142 (PMC8601520; doi:10.1371/journal.pone.0260142)
Supplement: Attachment — Submitted filename: Response to Reviewers.docx [file pone.0260142.s007.docx]

**REBUTTAL LETTER**

Dear editors and reviewers.

First and foremost, we want to express our deep gratitude to editors and reviewers who gave us valuable and practical advice. We carefully read your comments and revised our manuscript. Our responses are written in blue color.

There is being a new outbreak of the COVID-19 pandemic in Vietnam with more than 12,000 patients per day. We hope that editors, reviewers, and their families will be always in good health.

Thank you so much for your assistance.

Best regards.

Dai Xuan Dinh

**......................................................................................................**

**Journal requirements:**

1. Please ensure that your manuscript meets PLOS ONE's style requirements, including those for file naming. The PLOS ONE style templates can be found at

https://journals.plos.org/plosone/s/file?id=wjVg/PLOSOne_formatting_sample_main_body.pdf and

https://journals.plos.org/plosone/s/file?id=ba62/PLOSOne_formatting_sample_title_authors_affiliations.pdf

We strived to follow these templates.

2. Please include in your Methods section (or in Supplementary Information files) the participating hospitals/institutions.

Information on surveyed main hospitals and health facilities was added in the S2 Table and Method section (lines 136 - 147).

3. Please provide additional details regarding participant consent. In the ethics statement in the Methods and online submission information, please ensure that you have specify:

a) whether the ethics committee approved the verbal/oral consent procedure,

b) why written consent could not be obtained, and

c) how verbal/oral consent was recorded. If your study included minors, please state whether you obtained consent from parents or guardians in these cases. If the need for consent was waived by the ethics committee, please include this information.

The verbal consent procedure was approved by the ethics committee of Hanoi University of Pharmacy. The ethics committee did not require the research team to gain written consent from interviewees because in this study, we did not do clinical trials, did not take blood samples, and did not do any activities which could harm interviewees in any form. We only asked pharmacists about the availability and prices of medicines and therefore, it seems that the data-collection process did not have any detrimental effects on interviewees.

Not that written consent could not be obtained. From the beginning, we did not intend to obtain written consent. All public health facilities and private medicine outlets in our research are managed by the Hanam Department of Health. As a result, when data collectors gave a letter of endorsement (from the Hanam Department of Health) to interviewees (pharmacists), they were willing to participate in our research and answered questions about the availability and prices of essential medicines with pleasure. If there is anyone who did not want to take part in our research, we could not collect the data of their facilities.

**Reviewer 1**

**1. Introduction**

In lines 66-67, you stated several studies on the matter has been conducted; so what makes your different and urgent (is that because previous woks were unpublished?) what does it mean “small-scale studies”? (Lines 67 and 68)

We adjusted these sentences. The new information can be seen in lines 89 - 95. In the process of seeking previous studies involving essential medicines in Vietnam, we found several studies (written in the Vietnamese language). “Small-scale studies” in the first manuscript means in these studies, researchers only surveyed private medicine outlets (a study conducted in 2016), the low sample size - 14 public health facilities(a study conducted in 2014)... All these studies were conducted before the year 2017.

.....................................................................................................................................................

The introduction part does not introduce pharmaceutical delivery system of the Vietnam (Good to know here the current situation regarding healthcare in Vietnam, e.g. how is healthcare typically provided. What are the current co-payments for visiting a physician, e.g. 100%, 50%, etc., and what is this amount relative to the daily wage of the LPGW? What about medicines - are these 100% co-pay - some medicines are free for the public in the public sector and covered through insurance - what are these medicines and how are they chosen? ….do public hospitals and primary healthcare centers stock and dispense similar items? Are all drug outlets in private sectors drug stores and possess all types of EMs that public hospitals and primary healthcare centers can—if there is level of capacity of handling medicine?...), challenges to achieve this paper’s interest, reasons that trigger this work, and previous studies findings.

We did endeavor to supply information as much as possible.

The introduction to the Vietnam healthcare system can be seen in lines 64 - 91.

**2. The method**

Why was the Hanam chose over other potential sources in Vietnam?

How many districts are there in Hanam, and how are these six districts selected?

The introduction to the Hanam province and the reasons explaining why this province was chosen for investigation were added (lines 114 - 131). Hanam is a small province that is contiguous to the Hanoi capital. This province includes one city and five districts. All six areas were selected for investigation. All areas met the requirements of the WHO/HAI.

.....................................................................................................................................................

Are all the medicines included in this study expected to be equally available in hospitals, primary healthcare centers and drug stores due to legal restriction? If not, this may give wrong information about the availability of the medicines.

For the private sector, all medicines included in this study were expected to be equally available in private medicine outlets. All essential medicines are allowed to be stocked and sold in medicine outlets.

For the public sector, several medicines were only expected to be available in high-level hospitals/health centers (There are three levels: provincial, district, and commune). These medicines can be seen in the column “Levels of care” in the sheet “Reference prices” from the Workbook (S1 Data file). For these medicines, their availability was automatically computed and adjusted by the WHO/HAI workbook. For example, the level-of-care of insulin was 2. It means this medicine was only expected to be available in provincial and district health facilities (8 facilities). In our study, this medicine was available in only one public health facility (originator brand - OB). Therefore, the availability of OB insulin was equal to 1/8 (12.5%), in lieu of 1/30.

.....................................................................................................................................................

Since you are informing your study objective, how do you obtain/trace/ real patient price data on medicines especially from private sectors?

Interviewees (pharmacists) were only informed that some researchers would go to health facilities to do a scientific study (informed by leaders of the Hanam Department of Health and leaders of district health centers). We only informed interviewees about our study objective when we paid a visit to surveyed facilities to collect data.

The patient prices were taken from the labels of medicine containers (boxes) or computers. As per the Vietnam Pharmacy Law, all medicines in any medicine outlet must be labeled with a price tag on the boxes. The Department of Health periodically inspects medicine outlets. If there is any medicine without the price on the box, the owner will be fined. In the process of data collection, we did not observe any drugstores possessing a medicine without the price on the box. In some big medicine outlets and public health facilities, medicine prices were stored on the computers and we collected these prices from the computers.

.....................................................................................................................................................

For patient prices, MPRs should be lower than 1.5 in the public sector, and lower than 2.5 in the private sector. (Lines 129-130) Is there an agreed cut-point?

In many previous studies, these cut-off points were used to assess the suitability of medicine prices. For example:

- Gelders S, Ewen M, Noguchi N, Laing R. Price, availability and affordability: an international comparison of chronic disease medicines. World Health Organization and Health Action International; 2006. (Reference 22).

- Tadesse T, Abuye H, Tilahun G. Availability and affordability of children essential medicines in health facilities of southern nations, nationalities, and people region, Ethiopia: key determinants for access. *BMC Public Health*. 2021;21(1):714. doi: 10.1186/s12889-021-10745-5. PMID: 33849513; PMCID: PMC8045262. (Reference 46)

- Jiang M, Yang S, Yan K, Liu J, Zhao J, Fang Y (2013) Measuring Access to Medicines: A Survey of Prices, Availability and Affordability in Shaanxi Province of China. PLoS ONE 8(8): e70836. https://doi.org/10.1371/journal.pone.0070836

- ...

In our study, we used these cut-off points too.

.....................................................................................................................................................

The daily wages of the lowest-paid unskilled government worker in USD during data collection (lines 135-136)

Affordability limit/declaration cut-off this work?

The cut-off point was added (one day’s wage) in lines 192 - 194.

**3. Results**

Table 1 has to list individual medicines included in this study. The authors search continent approach to incorporate first column [Mean availability (standard deviation - SD)] in their new table. Such modification will give more detailed information about availability than the general one.

A table with the availability and MPRs of individual medicines was added (Table 1 in the revised manuscript).

.....................................................................................................................................................

Table 4. For price comparison, as the guideline, it is recommended to take medicines found at least four of drug outlets in each sites from both sectors. In current work OBs are absent in the public sector, no need to use them. The remaining LPGs found in both sectors has to be listed individually and their MPRs (Min, 25th %, median, 75th % and Max) included. Focus your comparative discussion on these drugs only. If possible, try to merge, Table 5 in to Table 4. Figures that will be listed in the revised Table 1 may show the rest.

The old Table 4 and Table 5 were merged into the new Table 5. This table has some general information on median MPRs of both OBs and LPGs in Hanam province and six areas so information on OBs was taken into account. Regarding special analyses, the median MPRs for medicines found as both product types (OBs and LPGs) can be seen in the S4 Table. The median MPRs for medicines found in both public and private sectors can be seen in the S5 Table.

.....................................................................................................................................................

Table 5 would particularly benefit if it is changed into Figure for better general analysis. Using your reference #13 patient price cut-off point, you can point out patient prices of selected medicine. There are now standard graphics (available from HAI) in which both availability and affordability are combined in one graph. The x-axis shows availability (ranging from 0-100%, with a vertical line at 80%. The y-axis shows the affordability, with a horizontal line at the chosen cut-off point. In this graph all products have a point-position. In an ideal world all products are located in the right-lower corner of the graph, with availability at >80% and affordability below cut-off. The graph can clearly visualize where the problems are.

(See these references for Figure <https://doi.org/10.1186/s12889-021-10745-5> and <https://doi.org/10.1371/journal.pone.0070836>)

Thank reviewer 1 for this recommendation. What a great idea! In the Results section, we added two Figures to comprehensively analyze the availability and prices of essential medicines. However, we still use MPRs to present the y-axis (similar to the two abovementioned references). Because only 12 medicines were used to compute affordability. MPRs were twice as much data as affordability. Therefore, using MPRs to present the y-axis can supply more information.

.....................................................................................................................................................

Table 6 would particularly benefit from an extensive review. In column 2, Drug name, strength, dosage form, dose, route of administration, frequency & treatment duration, Colum 3, Treatment schedule, The total amount of drug required to cover the complete treatment regimen, Column 4, Average drug Price per Unit (USD), and Column 5, Number of day’s wage to pay for treatment. In addition, sate reasons/description why bisoprolol 5 mg cap/tab and captopril 25 mg cap/tab used for hypertension management. This briefly shows how Vietnam STG (standard treatment guideline) looks like and how closer/far to/from IPRs (cost wise).

The recommended design for Table 6 is great. However, we still want to employ the design of WHO/HAI to report affordability. The design of not only Table 6 but also other tables in our first manuscript was taken from the *Survey Report Template Instructions* of WHO/HAI (page 30). The Report Template can be seen in the line *Chapter 12: Reporting: Supporting materials* in this link: <https://haiweb.org/what-we-do/price-availability-affordability/collecting-evidence-on-medicine-prices-availability>

All 14 treatment courses used to compute affordability were taken from the standardized WHO/HAI Workbook (to easily compare among regions and countries). The indications of Bisoprol and Captopril were also taken from this Workbook. The original Workbook without data can be also seen in the above link.

We also check the indications of these two medicines in the document published by the Vietnam Ministry of Health in 2018 (Vietnamese National Drug Formulary):

<http://phcnhagiang.org.vn/photos/tai-lieu/duocthuquocgia20181.pdf>

In this document, hypertension is one indication for both of them.

**Reviewer #2:**

**Abstract**

- Result (line 33): insert the exact p value.

The exact p-value was added (line 32).

**Introduction**

Line 50: Insert reference.

Reference was added (line 50).

.....................................................................................................................................................

Line 66: ………There are several studies on medicine prices, availability and affordability conducted in Vietnam [11, 12]. Some small scale studies were carried out but not published……….

What then is the gap in knowledge that this current study aimed to fill? Does this suggest that these studies were not on Essential medicines

Some new information was added in lines 89 - 95.

In 1985, the first National Essential Medicines List was published by the Vietnam Ministry of Health. The newest version is the 7th National Essential Medicines List released in 2018. There are several studies on medicine prices, availability and affordability conducted in Vietnam before the year 2018. From the year 2018 to now, in Vietnam, there is no study conducted to survey the availability, prices, and affordability of two types of essential medicines: originator brand and lowest-priced generic. Therefore, there is an urgent need to research essential medicines in Vietnam.

**Materials and methods**

Lines 86/87: ………A licensed private drugstore closest to each of the selected public facilities was selected for the private sector………

The WHO/HAI methodology also provides for survey of 'Other' pharmacies which include those domiciled in private/organization owned hospitals. Does the province have these categories of hospitals? if yes, why were they not included in the survey? if no, a brief description of the health facility set up in Hanam would suffice.

The healthcare system of Vietnam can be divided into two sectors: the public sector and the private sector. Some information on Vietnam’s healthcare system can be seen in lines 64 - 91. The Hanam healthcare system (lines 114 - 125) is similar to the national healthcare system.

.....................................................................................................................................................

Line 90: write ‘30’ in words

The number “30” was written in words (line 149).

**Results:**

Table 3: Urban (11 drug stores) versus rural (24 drug stores) comparison

Does this suggest that most of the districts surveyed were in the rural area? A brief sentence regarding the geography of these areas should be added in the methods section

Most of the areas in Hanam province are rural areas. The introduction to Hanam province was added in the Method section: Surveyed areas and health facilities (lines 114 - 125).

.....................................................................................................................................................

S1 Table

It is suggested that these medicines be group according to their class of drug. For example: Antibiotics, Psychotropic, Narcotics, Anti-inflammatory, Analgesics, Asthma medicines, Antihypertensive, Antidaibetic etc

The classes of surveyed medicines were added into the S3 Table - The list of surveyed medicines.

**Discussion**

Line 215: …….fairly high (public sector 63.2%, private sector 47.9%)……….

Recast to reflect that availability of LPGs in private sector was fairly high but that in public sector was low (47.9%)

We revised this mistake (lines 292 - 294).

.....................................................................................................................................................

Line 283: Vietnamese government has implemented many national policies to increase the availability reduce medicine prices

The authors should stress the need for the enforcement of the price regulation in the private sector. Despite that fact that prices are generally lower than those of other countries, the private sector still sells OBs higher than the LPGs to the tune of 351.34%. Unless the LPGs are of reasonable quality and efficacy in treatment of the particular disease. Are there concerns for substandard medicines in Hanam?

Some information on the prices of medicines in private medicine outlets was add in lines 386 - 390.

In private medicine outlets, regarding medicine prices, the government only requires that all medicines stocking in any medicine outlet must be labeled with prices on the medicine containers (boxes). In order to monitor and control the quality of medicines, the Department of Health in each province periodically inspects and surveys private medicine outlets. Many samples of medicines are randomly selected and tested to assess their quality.
